# Effect of Alternative Preservatives on the Quality of Rice Cakes as Halal Food

**DOI:** 10.3390/foods10102291

**Published:** 2021-09-27

**Authors:** Jungmin Oh, Mina K. Kim

**Affiliations:** 1 Korea Food Research Institute, 245, Nongsaengmyeong-ro, Iseo-myeon, Wanju-gun 55365, Jeollabuk-do, Korea; jmoh@kfri.re.kr; 2 Department of Food Science and Human Nutrition, Jeonbuk National University, 567 Baekjedaero, Deokjin-gu, Jeonju-si 54896, Jeonbuk, Korea

**Keywords:** halal, alcohol alternatives, rice cake, tteok

## Abstract

The halal food market is steadily increasing. The use of alcohol for any reason is strictly prohibited in halal foods; however, ethanol is widely used as a preservative for commercial rice cakes (tteok). The purpose of this study was to investigate the use of natural substances as alternative preservatives for rice cakes. Four different solutions were tested: distilled water (control), ethanol, grapefruit seed extract (GSE), and a mixture of citric extracts and organic acids (MCO). We investigated the total plate count (TPC), yeast and mold counts, color, texture profile assays (TPA), and sensory evaluation. Significant reductions of 3.65 log CFU were observed in TPC in rice cake treated with MCO solution after 28 days of storage. However, mold and yeast counts were only reduced by ethanol treatment. Among the physical texture properties analysis, hardness was maintained for the 28 days in all samples. The total color difference values (ΔE) revealed no significant color changes in any rice cake compared to the controls. The ethanol-treated rice cake scored the lowest for overall preference and desired hardness.

## 1. Introduction

Halal food is defined in the Quran, the holy book of Islam, and essentially means food that is permissible according to Islamic law. Halal food laws strictly prohibit the use of haram (which literally means prohibited), meaning that foods containing haram animals and any form of alcohol should not be included in halal foods designed for Muslims [1,2]. Among haram, the term alcohol extends beyond ethanol (ethyl alcohol) to include any organic compound with a hydroxyl functional group (-OH) bound to a saturated carbon atom, such as methanol, propanol, butanol, pentanol, and hexanol [3]. However, ethanol is considered haram for Muslims because it causes intoxication.

Ethanol is the most common volatile compound in food produced by the fermentation of sugars and is widely used as an important organic solvent for flavorings, colorants, and preservatives in processed foods [4]. While ethanol can serve as a chemical food preservative, finding natural alternatives for ethanol for use as food preservatives in halal foods is necessary to ensure the safety of halal foods [5]. Various natural and naturally derived products have been tested for controlling the growth of pathogenic bacteria [6], and citrus extracts and organic acids have been reported to have effective antimicrobial activities.

Previous studies have reported the antibacterial activities of bitter orange (*Citrus aurantium*), also called Seville orange [7]. Bitter orange originated from India, and its oil, peel, flowers, and leaves are classified as Generally Recognized As Safe (GRAS) food additives in Korea [8]. A previous study on the antibacterial activity of *C. aurantium* flowers confirmed their efficacy against all tested gram-positive and gram-negative bacterial species [9]. Another study also reported the antimicrobial activities of citrus extracts against the biofilms of *Enterococcus faecalis* [10].

Antimicrobial activity against foodborne pathogens is well documented for organic acids, which are natural substances present in various fruits and fermented products [11]. Organic acids can suppress the growth of microorganisms by flowing through the cell membranes of the microorganisms and becoming ionized inside the membranes. The microbial cell must maintain the intracellular pH by releasing hydrogen ions, as the acidic pH inside the cell damages the extracellular membrane by modifying and damaging enzyme activity and protein and DNA structure [12]. The high pKa value of organic acids increases the chances of them being in their non-dissociated forms that are favored for crossing cell membranes [13]. Changes in the permeability of the cell membrane hinder substrate transport, while pH changes inside the cell suppress NADH oxidation, thereby affecting the electron transport system and leading to the death of the microorganism [14]. For this reason, the use of a mixture of organic acids with different pKa values could be more advantageous than a single organic acid alone for food preservation [13].

One traditional preserved food in Korea is rice cake, which is made of rice flour and consumed as a meal or snack [15]. Korean rice cakes are gaining popularity in Islamic countries, but the use of ethanol as a preservative renders them haram. The typical method for preparing rice cake involves steaming a rice flour and water mixture and adding sugar or salt according to preference [16]. The steaming step causes the starch component to undergo retrogradation [17,18], with storage temperature and time commonly affecting the rate of retrogradation, with a maximum value at 5 °C [19]. Consequently, food products made from cereal grains can become hazardous, particularly due to contamination by *Salmonella* spp., *Staphylococcus aureus*, and *Bacillus cereus* [20]. *Bacillus cereus* (gram-positive), in particular, has been identified worldwide in outbreaks associated with cooked rice [21].

In Korea, 19.3% of the rice cakes with filling and 19.3% of the rice cakes without filling were reported to be contaminated with *S. aureus* [22]. The high moisture content and high water activities (AW over 0.85) have led to several foodborne outbreaks related to rice cake. In 2010, an investigation of microbial contamination in over 30 rice cake items in Korean retail stores revealed total microorganism counts of 2.36–6.89 log CFU/g, coliform counts of 1.32–4.69 log CFU/g, and *B. cereus* counts of 1.38–2.48 log CFU/g [23]. For these reasons, efforts must be made to ensure the microbiological safety of rice cakes. The common industrial practice in Korea is to use ethanol as a microbial preservative by spraying or briefly soaking rice cake before packaging [1]. However, this use of ethanol limits the permissibility of these rice cakes for consumption by persons following halal food diets. Therefore, the objective of this study was to investigate the use of natural antimicrobial agents that could replace ethanol as a preservative in halal food. We evaluated quality changes in rice cake by microbial analysis (total plate count and mold and yeast counts) and color and texture tests at 0, 7, 21, and 28 days after treatment. Sensory quality was determined based on a sensory panel evaluation at 8 and 35 days.

## 2. Materials and Methods

### 2.1. Sample Preparation

Rice cakes (Cheiljedang, CJ, Seoul, Korea) were purchased directly from the manufacturer to allow testing of the same batch of product. The rice cakes were divided into four random groups for treatment with four different antimicrobial solutions: distilled water (control), 70% ethanol (Hanson Hygiene, Cheonan, Korea), 1% grapefruit seed extract (GSE; Quinabra-Quimica Natural Brasilier Ltd., São José dos Campos, SP, Brazil), and a 1% mixture of citrus extracts and organic acids (MCO), consisting of 72% *Citrus aurantium* (bitter orange), 1% ascorbic acid, 1% lactic acid, 2% citric acid, and 24% distilled water (Seoul Food R&D Co., Ltd. Seongnam, Korea). The samples were soaked in each solution for one minute and then dried for 5 min at room temperature to remove excess solution left on the rice cake surface. The samples were then vacuum-sealed in polyethylene (PE) bags using a vacuum sealer (V4800 Vacuum Sealing System, FoodSaver, Atlanta, GA, USA) and stored for 35 days at refrigerator temperature (4 °C).

### 2.2. Microbial Analysis

Microbial analyses, including total plate counts and yeast and mold counts, were performed using Petrifilm plates (3M^TM^, Minnesota, USA) following a standard method of analysis. Briefly, 1 mL of appropriate diluent was plated onto the plate and incubated in a dry oven (HSD-150, Sinan Science Industry Co., Ltd., Gyeonggi, Korea) at 37 °C for 48 h (aerobic count plates) or at 25 °C for 12 h (yeast and mold count plates). Microbial analysis was conducted at 0, 7, 21 and 28 days of storage. All microbial analyses were performed in triplicate on three independent batches of rice cake.

### 2.3. Color Assessment

Color measurement was performed using a colorimeter (Color reader CR-400, Konica Minolta Sensing Inc., Tokyo, Japan) to assess any potential color changes attributable to the antimicrobial solutions. Before measuring the sample color, the colorimeter was calibrated with a white plate. Color assessments were performed on the rice cake surface. The rice cake was prepared by being cut into 1 cm^3^ pieces. All measurements were taken from five randomly chosen surfaces of each sample. The experiments were replicated 15 times. L* (lightness), a* (red to green), b* (yellow to blue), and Δ*E* (total color difference) were reported. The average values of L*, a*, and b* were used to calculate Δ*E* as follows:

$$\Delta E={\sqrt{\left(L^{*}-L^{c}\right)}}^{2}+{\sqrt{\left(a^{*}-a^{c}\right)}}^{2}+{\sqrt{\left(b^{*}-b^{c}\right)}}^{2}$$

### 2.4. Texture Profile Analysis (TPA)

The hardness property is a major textural parameter for the evaluation of the storage of rice cake [24]. Commonly, rice cake is reheated before eating. For the texture profiles, the rice cake was cut into 1 cm^3^ pieces and then heated in boiling water for 45 s. Following boiling, the rice cake samples were immediately placed in a desiccator for 3 min to cool. The texture profile analysis was performed on the rice cake after 0, 7, 21, and 28 days of storage using a TA-XT Plus 50 texture analyzer (Stable Micro System Ltd., Surrey, UK). The test was conducted using a 35-mm cylindrical aluminum plate at a pre-test speed of 3.00 mm/s, test speed of 1.00 mm/s, and post-test speed of 1.00 mm/s at 40% strain. The hardness of the rice cake was determined from the time–force curve. The experiments were replicated 15 times.

### 2.5. Consumer Acceptance Test

The development of any flavor/aroma derived from the treatment solution was evaluated by consumer acceptance tests conducted twice (on day 8 and day 35) with 43 panelists who had more than 5 years of experience in the sensory evaluation of food products. Two different time points were selected for consumer testing to measure consumer acceptance at the beginning of storage (day 8) and at the end of storage (day 35), based on the typical shelf life of commercially available rice cake (30 ± 10 days in the refrigerator).

The sample preparation for sensory evaluations was as follows. Prior to the evaluation, the rice cake was removed from the refrigerator, the packaging was removed, and the cakes were cut into pieces 1 cm in radius × 3 cm in height. The rice cake was blanched in hot water for 2 min before serving at 40 °C. The rice cake pieces were served on a 10 cm diameter white paper plate (Clean Wrap, Seoul, Korea), labeled with 3-digit random codes. Two pieces of rice cake for each treatment were presented to participants, and all four samples were presented at the same time.

The overall testing procedures were modified from a previous study [25]. When evaluating the samples, the panel members were asked to evaluate their opinions, such as overall liking and specific attributes, including appearance, smell/odor, taste/flavor, and texture (hardness), on a standard 9-point hedonic scale, ranging from “9 = like extremely” to “1 = dislike extremely”. A 10 s rest between sample evaluation was enforced to minimize the carry-over effect, and mineral water and plain crackers were provided to cleanse the palate during the rest time [26].

### 2.6. Ethanol Analysis

Confirmation that residual ethanol in the samples met halal criteria was obtained by quantitative analysis using an in-house method [27]. Sample preparation for gas chromatography-flame ionization detection analysis was as follows. Each rice cake was thoroughly ground up using a mixer (Büchi, Flawil, Switzerland). A sample of the powder (0.5 ± 0.01 g) was added to a gas-tight vial (Supelco, Bellefonte, PA, USA) containing 1-propanol (≥99.9%; Sigma-Aldrich Inc., MO, USA) as the internal standard. Ethanol was extracted with water (analytical grade; Daejung Chemicals and Metals Co., LTD., Gyeonggi, Korea) and stirred with a multi-point stirrer (Cimarec™ i Telesystem Multipoint Stirrers; Thermo Fisher Scientific LTD., Seoul, Korea) for 60 min at room temperature. After extraction, the supernatants were filtered through a Whatman 0.45 μm PTFE syringe filter (LK Lab Korea Co., LTD., Gyeonggi, Korea).

Quantitative analysis of the ethanol from each sample was conducted using a gas chromatograph (GC-2030 Shimadzu, Kyoto, Japan) equipped with an HP-INNOWAX column [30 m (L) × 0.25 mm (ID) × 0.25 μm film thickness; Agilent Technologies Inc. (Santa Clara, California, USA)] as a stationary phase. Each sample (1 μL) was injected in split mode with a split ratio of 13:1. The gas flow rates were maintained as follows: carrier gas (helium, 10 mL/min), hydrogen (30 mL/min), and air (300 mL/min). The injector and detector temperatures were maintained at 180 and 250 °C, respectively. The oven temperature was initially held at 50 °C for 1 min, then increased from 50 to 160 °C at a rate of 5 °C/min, and held for 2 min.

### 2.7. Statistical Analysis

The results were expressed as mean ± standard error and were representative of experiments. Two-way ANOVA was performed in order to determine the effect of time and sample treatment (solution), as well as the interaction between time and solution. The interaction term (time × solution) was evaluated prior to determine the main effect (time effect and solution (treatment) effect), and no significant difference was found in the interaction term (time × solution). The data were statistically analyzed using IBM SPSS Statistics version 20. The significance level was established as 0.05.

## 3. Results and Discussion

### 3.1. Microbial Analysis

Table 1 shows the antimicrobial effect of each treatment on the total plate counts (TPCs) and yeast counts from the rice cake. Regardless of the treatment, the populations of microorganisms increased over time. On Day 0, the rice cake treated with ethanol had the lowest total plate counts among the samples, indicating an immediate antimicrobial activity of ethanol. Notably, the MCO-treated rice cake had the lowest total plate counts at 28 days. GSE is well known to act as an effective natural antimicrobial agent. Compared to GSE, the MCO-treated rice cake reduced the TPCs by 3.05 log CFU/g (day 28). The ethanol-treated rice cake showed the lowest microbial count at day 0 among all samples, but the MCO-treated rice cake showed the least microbial growth after 28 days of storage, confirming MCO as an effective antimicrobial agent during storage. After 21 and 28 days of storage, the ethanol-treated and MCO-treated rice cake had significantly different TPC values compared with the distilled water-treated and GSE-treated rice cake (*p* < 0.05). At the last day of storage (day 28), the TPC values were 6.22 ± 0.21 log CFU/g (distilled water), 4.05 ± 1.31 log CFU/g (ethanol), 6.70 ± 0.55 log CFU/g (GSE), and 3.65 ± 0.09 log CFU/g (MCO) (*p* < 0.05). These TPC values supported the use of MCO as a substitute preservative for ethanol.

No yeast or mold colonies were found in any of the rice cake samples at day 0, regardless of treatment. The yeast and mold counts increased more slowly than the TPCs and remained low during the storage. Yeast and mold counts did not increase in the ethanol-treated rice cake until day 7, and a slight increase (0.82 ± 1.42 log CFU/g) was observed on day 21. Rice cake treated with MCO followed a similar pattern to that of ethanol until day 7, but a slight increase in yeast and mold counts was observed later (Table 1). At 28 days, MCO showed values of 4.19 log CFU/g for yeast and mold, but this value was still within the acceptable range for yeast and mold counts established by the Korean Food Standard Codex (less than 4.7 log CFU/g). Yeasts and molds are unable to form colonies in anaerobic conditions, but our vacuum-sealed packages were unable to create perfectly anaerobic systems, and some air could leak through the outer layer of the film. Multiple layers, thicker packaging, or modified atmosphere packaging could be used to ensure full restriction of oxygen. Based on these results, the use of MCO preservatives for halal rice cakes might require more complex packaging.

### 3.2. Color Assessment

The color changes during storage of the rice cake treated with different solutions are shown in Table 2. One of the most important attributes considered by consumers is food color, as this is associated with food quality [28]. The ideal color of rice cake is white, with a high lightness value [29]. At 28 days, the L* values of all samples had decreased naturally from the value at day 0, indicating a decline in the lightness value during rice cake storage. The rice cakes treated with MCO and ethanol showed significantly higher values than the control and GSE-treated rice cakes, indicating that MCO and ethanol preservatives retained a better lightness quality than the other treatments. The addition of preservatives could have resulted in yellowing or darkening of the rice cake color due to chemical changes; however, the total color difference values (Δ*E*) indicated that the colors of all the treated rice cake samples were not significantly different from the control. Therefore, none of the preservative solutions affected the color of the rice cake.

### 3.3. Texture Profile Analysis (TPA)

**Figure 1:**
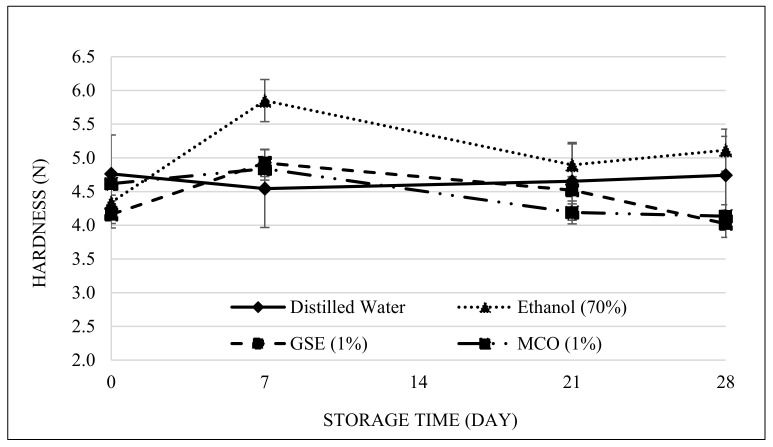
Changes in hardness (N) of rice cakes that were treated with distilled water, ethanol (70%), GSE (1%), or MCO (1%) (*p* < 0.05).

Investigation of the rice cake hardness for 28 days (Figure 1) revealed a significantly lower hardness for the MCO-treated rice cake (4.14 N) than for the 70% ethanol-treated rice cake (5.11 N) (*p* < 0.05). The hardness of the MCO samples increased slowly until the last storage day. The hardness of a rice cake is a typical macroscopic aspect that determines starch retrogradation [30] and is a main factor indicating decreases in rice cake quality during storage. According to these data, retarding the firming rate after treatment would make MCO a suitable preservative agent for rice cake. A previous study has reported that moisture reduction in rice cake affects the hardness, as indicated by changes in the hardness of rice cake samples after 4 days of storage (5.41 N) [31]. In the present study, all samples maintained their hardness quality after 28 days, indicating that vacuum-sealed packaging might preserve rice cake dehydration.

### 3.4. Consumer Acceptance

The marketability of rice cakes preserved with different preservatives was assessed by consumer acceptability tests (Table 3). Consumer acceptance testing results after 7 days of storage showed significant differences in the overall liking scores among the rice cakes treated with different preservative agents (*p* < 0.05). The rice cake treated with ethanol received significantly lower overall liking scores (4.77) than the other samples (*p* < 0.05). No significant differences were noted for the other three treatments: control (5.70), GSE (5.64), and MCO (5.61) (*p* > 0.05). Similar to the overall liking score, the ethanol-treated sample received a lower flavor liking score (4.00) than the others (*p* < 0.05), while no significant differences were noted in flavor likings for the other samples: control (4.70), GSE (5.09), and MCO (4.55). Even after the rice cake was boiled for 2 min, some of the panelists could still smell a strong alcoholic odor, and this may have led to the decrease in overall liking and flavor liking of the rice cake treated with ethanol. A previous study in our halal lab at the Korea Food Research Institute also indicated that Muslims tend not to buy food with an alcohol odor (for example, food treated with ethanol as a preservative), even if it meets the halal regulations.

Similar results were demonstrated after 35 days of storage, as the overall liking score was significantly lower for the ethanol-treated rice cake (5.09) than for the other samples (*p* < 0.05), while no significant differences were seen among the other three samples (*p* > 0.05): control (6.26), GSE (6.12), and MCO (6.28). For flavor liking, the ethanol sample again received the lowest flavor liking score (4.00) compared to the other samples (*p* < 0.05). The flavor liking score after 35 days also followed a similar trend to the results at day 7. The score for the ethanol-treated sample was 4.47, which was significantly lower than the scores for the other samples (*p* < 0.05). No significant difference was seen among the other three samples (*p* > 0.05): control (6.23), GSE (5.77), and MCO (6.05). For hardness liking, the ethanol-treated rice cake again received the lowest hardness liking (5.51) compared to the control sample (6.53; *p* < 0.05).

Open questions were also included to determine the specific reasons for the consumers’ likes and dislikes for each sample. In total, 16.27% of the participants responded that a unique aroma was released from the ethanol-treated rice cake. All rice cake samples were soaked for the same time with the preservative agents and were washed and boiled under identical conditions. Even with these preparations, only the ethanol treatment carried over an alcoholic odor and taste to the rice cake. These factors might impart a negative taste sensation to consumers. For natural antimicrobial agents, GSE is the most popular agent and is widely used in the food industry, despite a disadvantage imparted by the bitter taste of GSE [32]. However, in the present study, no negative aspects were discerned with the use of GSE as a rice cake preservative.

### 3.5. Ethanol Analysis

The analytical method in this study was applied to determine the ethanol content of the rice cake treated with different preservative agents. Only ethanol was detected from the ethanol-treated rice cake (0.71 ± 1.76 mg/g). The ethanol content determined in those samples was compared with the threshold of permissible ethanol in halal foods established by two major Halal Certification Bodies (JAKIM; Malaysia, MUIS; Singapore). The JAKIM permissible ethanol level in halal foods is less than 0.5% remaining in the final product. The MUIS permissible ethanol level is much stricter, at less than 0.1% remaining in the final product [33]. According to halal regulations and standards, our ethanol-treated rice cake would not obtain a halal food certification, but MCO and GSE could be used as halal food preservatives.

## 4. Conclusions

This study evaluated the efficacy of a newly developed mixture of citrus extracts and organic acids (MCO) to improve the physicochemical properties and microbial stability of rice cake. Our analysis of various characteristics, such as microbial analysis, hardness, color value, and consumer liking, revealed that MCO restricted and retarded microbial organism growth over a 28-day shelf life. In addition, the MCO left no alcoholic odor or taste, which is a main issue among manufactured rice cakes. MCO treatment also did not significantly affect the perceived sensory characteristics of rice cake; therefore, MCO preservatives may not negatively affect consumer acceptance of the treated products. Lastly, MCO samples met the halal regulations regarding ethanol content. Therefore, the MCO preservatives could be a useful alternative for use in the halal food industry. Further research is needed on other processed foods to generalize the efficacy of this non-ethanolic antimicrobial agent and its application to halal foods. The MCO preservative was investigated only using total plate counts and mold and yeast counts in the present study. Tests of the inhibitory activity against target toxigenic strains are still needed to verify the safety of using MCO as a rice cake preservative.

## Figures and Tables

**Table 1:** Total plate counts and mold and yeast counts of rice cake during storage.

Treated Solution	Total Plate Count (log CFU/g)				Mold and Yeast Count (log CFU/g)			
	0 Day	7 Day	21 Day	28 Day	0 Day	7 Day	21 Day	28 Day
Distilled Water	4.61 ± 0.08 c,d	5.26 ± 0.10 d,e	6.14 ± 0.13 b,f	6.22 ± 0.21 b,f	0.00 ± 0.00 d	2.78 ± 0.06 b,e	4.34 ± 0.63 b,f	3.89 ± 0.16 b,f
Ethanol (70%)	2.85 ± 0.66 a,d	2.58 ± 0.04 a,d	4.10 ± 0.16 a,e	4.05 ± 1.31 a,e	0.00 ± 0.00	0.00 ± 0.00 a	0.82 ± 1.42 a	0.83 ± 1.44 a
GSE (1%)	4.31 ± 0.26 b,c,d	4.91 ± 0.24 c,d	6.41 ± 0.04 b,e	6.70 ± 0.55 b,e	0.00 ± 0.00 d	3.09 ± 0.13 c,e	3.75 ± 0.02 b,e,f	4.49 ± 0.60 b,f
MCO (1%)	3.81 ± 0.12 b,d	3.86 ± 0.00 b,d	4.21 ± 0.73 a,e	3.65 ± 0.09 a,e	0.00 ± 0.00 d	0.00 ± 0.00 a,d	4.19 ± 0.84 b,e	4.19 ± 0.05 b,e

Different letters indicate significant differences (*p* < 0.05) between mean values in column (a, b, c) and rows (d, e, f).

**Table 2:** Color value of rice cake during storage: L*, lightness; a*, redness; b*, yellowness; Δ*E*, total color difference values.

Value	Treated Solution	Storage Day			
		0 Day	7 Day	21 Day	28 Day
L*	Distilled Water	78.53 ± 1.99 b,c,h	69.22 ± 1.51 a,g	68.95 ± 1.19 b,g	69.99 ± 1.61 c,g
	Ethanol (70%)	77.25 ± 1.65 a,h	69.07 ± 1.35 a,g	67.10 ± 1.68 a,f	67.67 ± 1.07 a,f
	GSE (1%)	78.95 ± 1.30 c,g	68.98 ± 1.18 a,f	69.28 ± 1.51 b,f	69.21 ± 1.74 b,c,f
	MCO (1%)	79.20 ± 1.08 c,h	68.63 ± 1.49 a,f,g	69.44 ± 1.04 b,g	68.59 ± 2.14 a,b,f,g
a*	Distilled Water	1.97 ± 0.10 b,h	1.55 ± 0.07 c,g	1.47 ± 0.05 c,f	1.56 ± 0.12 c,g
	Ethanol (70%)	1.81 ± 0.10 a,i	1.55 ± 0.07 c,h	1.37 ± 0.08 b,g	1.44 ± 0.12 b,g
	GSE (1%)	1.82 ± 0.10 a,i	1.37 ± 0.07 a,h	1.27 ± 0.10 a,f,g	1.34 ± 0.13 a,g,h
	MCO (1%)	1.93 ± 0.07 b,i	1.66 ± 0.06 d,h	1.41 ± 0.06 b,f	1.41 ± 0.08 a,b,f
b*	Distilled Water	0.78 ± 0.71 a,f	0.88 ± 0.32 a,f	0.59 ± 0.21 a,f	0.67 ± 0.48 a,f
	Ethanol (70%)	1.44 ± 0.48 c,g	0.92 ± 0.55 a,f	1.20 ± 0.19 d,f,g	1.36 ± 0.31 c,g
	GSE (1%)	1.06 ± 0.40 a,b,c,f	1.34 ± 0.53 b,f,g	1.52 ± 0.18 e,g	1.05 ± 0.47 b,f
	MCO (1%)	1.34 ± 0.54 b,c,g	0.84 ± 0.47 a,f	0.81 ± 0.16 b,f	0.72 ± 0.24 a,f
Δ*E*	Distilled Water	19.88	28.98	29.23	28.12
	Ethanol (70%)	21.29	29.13	31.15	30.53
	GSE (1%)	19.56	28.39	29.09	28.98
	MCO (1%)	19.38	28.64	28.80	29.52

Different letters indicate significant differences (*p* < 0.05) between mean values in column (a, b, c, d, e) and row (f, g, h, i).

**Table 3:** Consumer acceptance test result of rice cake with different preservative agents.

Treated Solution	7 Storage Day				35 Storage Day			
	Distilled Water	Ethanol (70%)	GSE (1%)	MCO (1%)	Distilled Water	Ethanol (70%)	GSE (1%)	MCO (1%)
Overall liking	5.70 ± 1.47 a	4.77 ± 1.5b	5.64 ± 1.45 a	5.61 ± 1.67 a	6.26 ± 1.65 b	5.09 ± 1.76 a	6.12 ± 1.35 b	6.28 ± 1.50 b
Color liking	6.11 ± 1.47 *	6.32 ± 1.64 *	6.27 ± 1.53 *	6.20 ± 1.37 *	6.58 ± 1.38 *	6.14 ± 1.47 *	6.63 ± 1.18 *	6.72 ± 1.26 *
Flavor liking	4.70 ± 1.96 a, b	4.00 ± 1.63 a	5.09 ± 1.78 b	4.55 ± 1.82 a, b	6.23 ± 1.69 b	4.47 ± 2.14 a	5.77 ± 1.49 b	6.05 ± 1.76 b
Taste liking	5.50 ± 1.87 *	4.80 ± 1.87 *	5.59 ± 1.67 *	5.25 ± 1.62 *	6.14 ± 1.91 b	4.98 ± 2.04 a	5.86 ± 1.58 b	6.05 ± 1.80 b
Hardness liking	5.93 ± 1.82 *	5.48 ± 1.80 *	6.05 ± 1.60 *	5.89 ± 1.78 *	6.53 ± 1.71 b	5.51 ± 1.92 a	5.79 ± 1.91 a, b	6.06 ± 1.86 a, b

Consumer acceptance was measured on a 9-point hedonic scale with 1 = “extremely dislike” and 9 = “extremely like.” The values represent the mean ± standard deviation of liking scores collected from 43 consumers. Different letters indicate significant differences (*p* < 0.05) between mean values in column (a, b) and * mark in a row within the same storage day represent no significant differences among samples (*p* < 0.05).

## Data Availability

Data is contained within the article.
